# Massive Rotator Cuff Repair: Banana SutureLasso Double-Row Fixation

**DOI:** 10.1016/j.eats.2020.10.048

**Published:** 2021-02-18

**Authors:** Anthony Kamel, Marie Caroline Merlet, Franck Dujardin, Matthieu Lalevée, Olivier Courage

**Affiliations:** a Havre Arthroscopy Surgery School, Hôpital Privé de l’Estuaire Groupe Ramsay Générale de Santé, Le Havre, France; b Centre Hospitalier Universitaire de Rouen, Service de Chirurgie Orthopédique et Traumatologique, Rouen, France

## Abstract

Massive rotator cuff tears have always been a worrisome situation to every orthopaedic surgeon. Patients’ functional demands are increasing with time, and this is why we aim to offer them the best options to preserve their quality of life. We found that using the Banana SutureLasso (Arthrex) has made this type of surgery much easier. We think that with the Banana SutureLasso (Arthrex) we have more access to the medial part of the rotator cuff, and that we can grab both the deep and superficial layers of the tendons while diminishing the risk of laceration. We combined an X-suture with a double-row fixation using a Corkscrew (Arthrex) on the humeral tuberosity and a SwiveLock (Arthrex) screw on lateral side of the humerus. We used only 3 portals with an extra parking portal. Another advantage is that we can adjust the sutures so they would be perpendicular to both ends of the tear. Our technique is simple, safe, and reproducible.

Arthroscopic massive rotator cuff repair is always considered a challenging surgical procedure. It is defined as a tear exceeding 5 cm or involving 2 or more cuff tendons.^1^ Arthroscopic procedures are increasingly becoming the gold standard treatment of these tears for its advantages that include a minimally invasive technique with lower morbidity and a faster recovery than open repair procedures. Numerous techniques have been published and used to treat and repair these tears; these include single- or double-row fixations, sometimes using auto- or allograft for irreparable tears, showing very promising results.^2^ Although no significant clinical differences between single- and double-row techniques were shown in most recent studies,^3^^,^^4^ controversy still exists on the treatment of massive rotator cuff tears. Thorsness and Romeo^2^ found that superior capsular reconstruction are the best solution for irreparable cuff tears. The most challenging part while repairing a massive rotator cuff tear is tissue laceration while trying to reduce and suture the tear (Table 1). This is why we found that using the Banana SutureLasso (Arthrex) provides access to the most medial part of the cuff to have a better hold on the tissue, therefore decreasing the risk of failure related to laceration. Our technique involves a simple X-suture using the Banana SutureLasso (Arthrex), then a double-row fixation using a Corkscrew (Arthrex) on the humeral tuberosity, and a SwiveLock (Arthrex) screw on the lateral side of the humerus.

**Table 1:** Advantages/Disadvantages

Advantages
• Sutures can be adjusted so they can be perpendicular to the axis of the tear
• Grabbing more tissue from the medial part, decreasing laceration risk
• Holding both the superficial and deep layers of the rotator cuff tissue
Disadvantages
• Learning curve of the technique
• Adjusting the wires at every step
• Finding the best entry point for the Banana SutureLasso (Arthrex)

## Patient Position and Approaches/Portals

**Fig 1:**
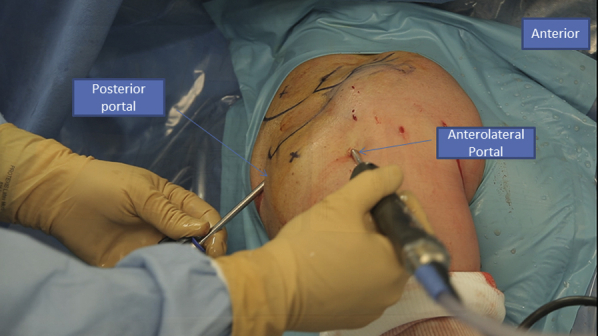
Right side, beach chair position: posterior and anterolateral portals used to explore the shoulder.

**Fig 2:**
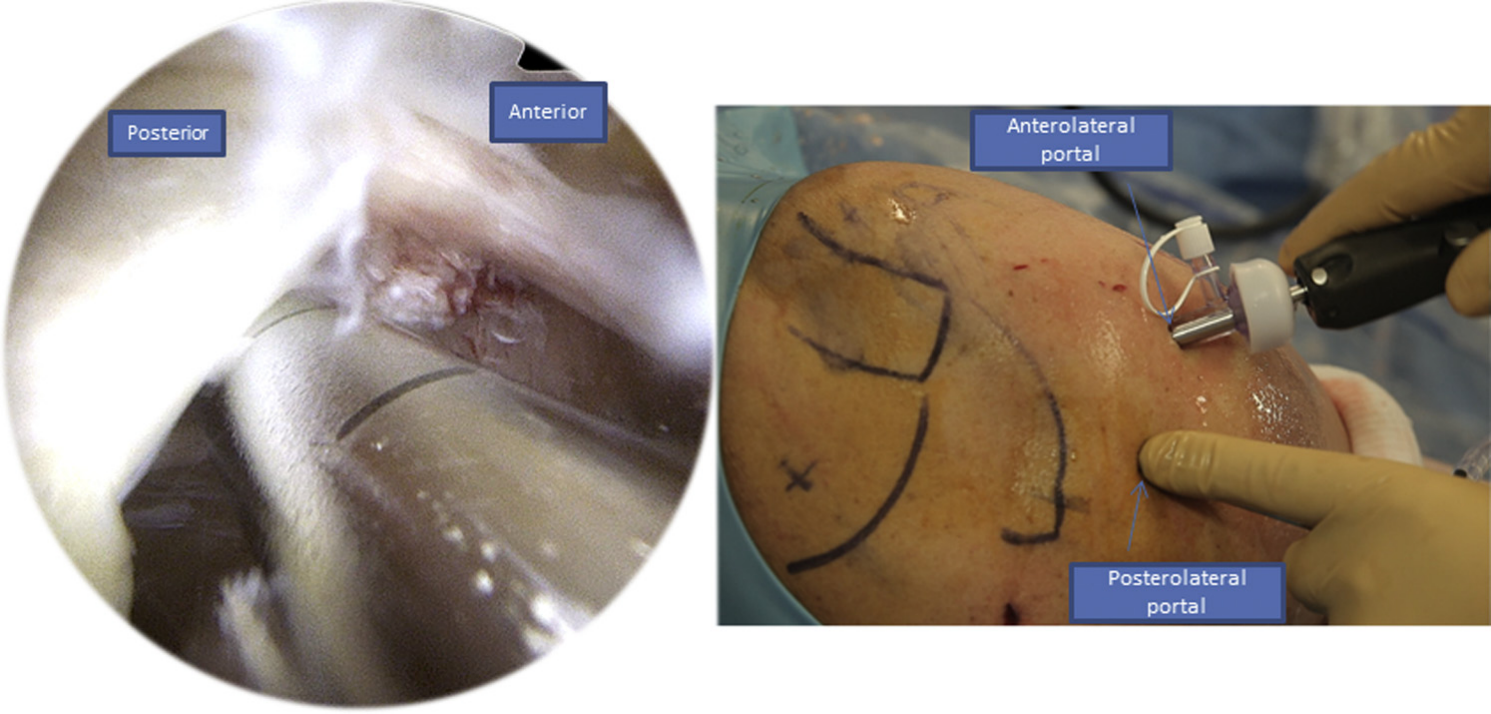
Right side, beach chair position, posterolateral portal for the arthroscope: in this portal we have a clear view of the tear.

The patient is in a classic beach chair position (Video 1). We begin with the intra-articular exploration using a posterior portal through the “soft point” to access the shoulder where the arthroscope is administered. An anterolateral instrumental portal is made for tenotomy/tenodesis of the long biceps (Fig 1). Then we proceed to the subacromial space using the same approaches. After bursectomy and subacromial exploration, we assess the rotator cuff tear. To do so, we add a posterolateral portal used for the arthroscope to face the tear and have a better exposure over the humeral head and the rotator cuff tissue (Fig 2).

## Surgical Technique

### First Part: The X-Suture Using the Banana SutureLasso

**Fig 3:**
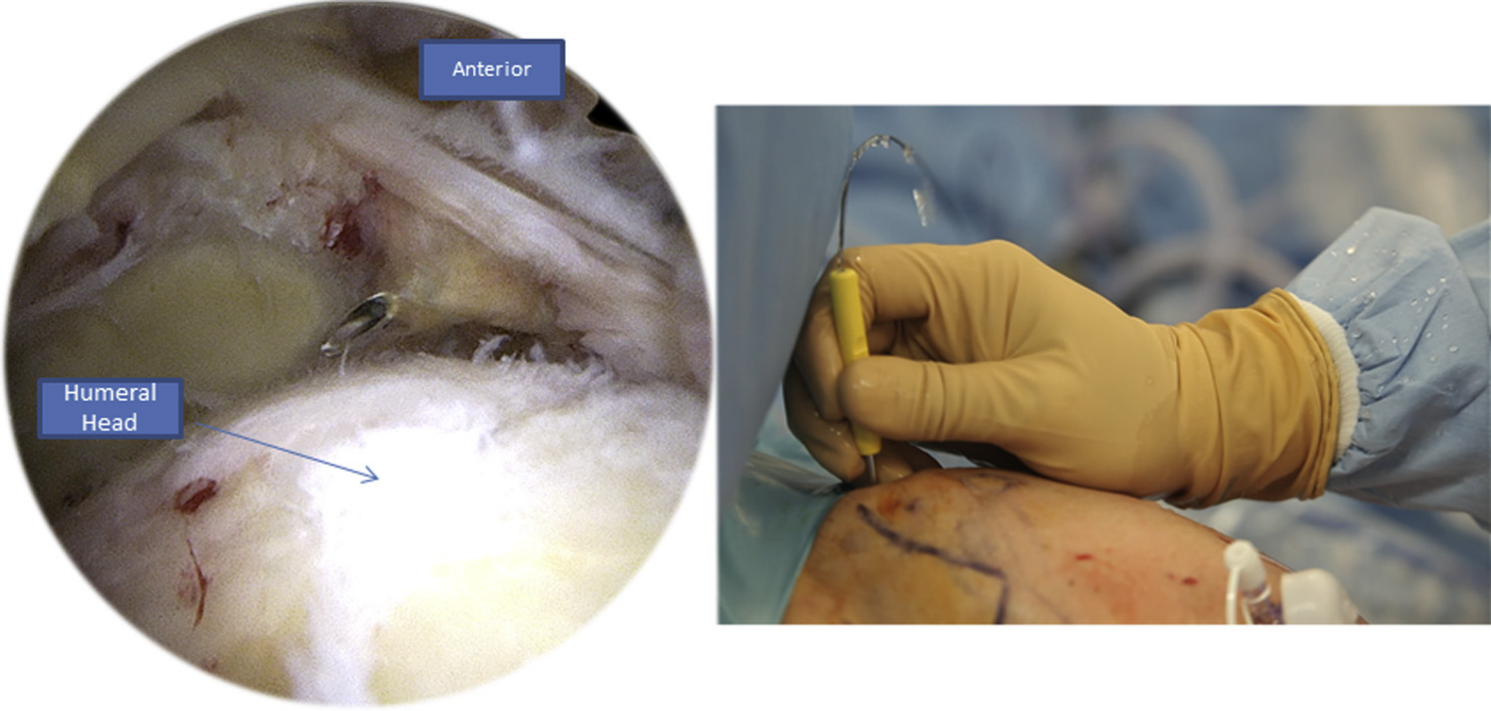
Right side, beach chair position, posterolateral portal for the arthroscope: the X-suture is begun with the introduction of the Banana SutureLasso (Arthrex) anteriorly.

**Fig 4:**
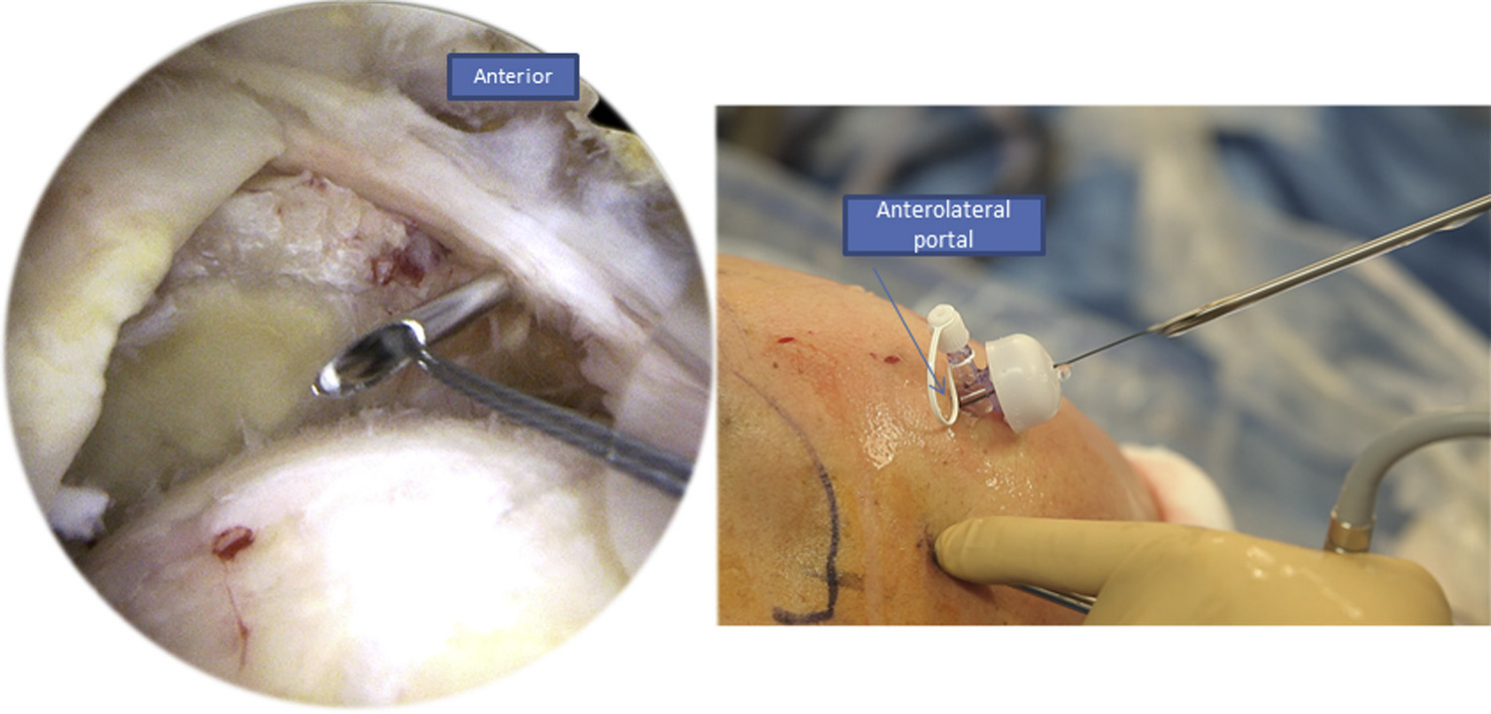
Right side, beach chair position, posterolateral portal for the arthroscope: to retrieve the FiberTape (Arthrex) the tracking wire is passed through the canula.

**Fig 5:**
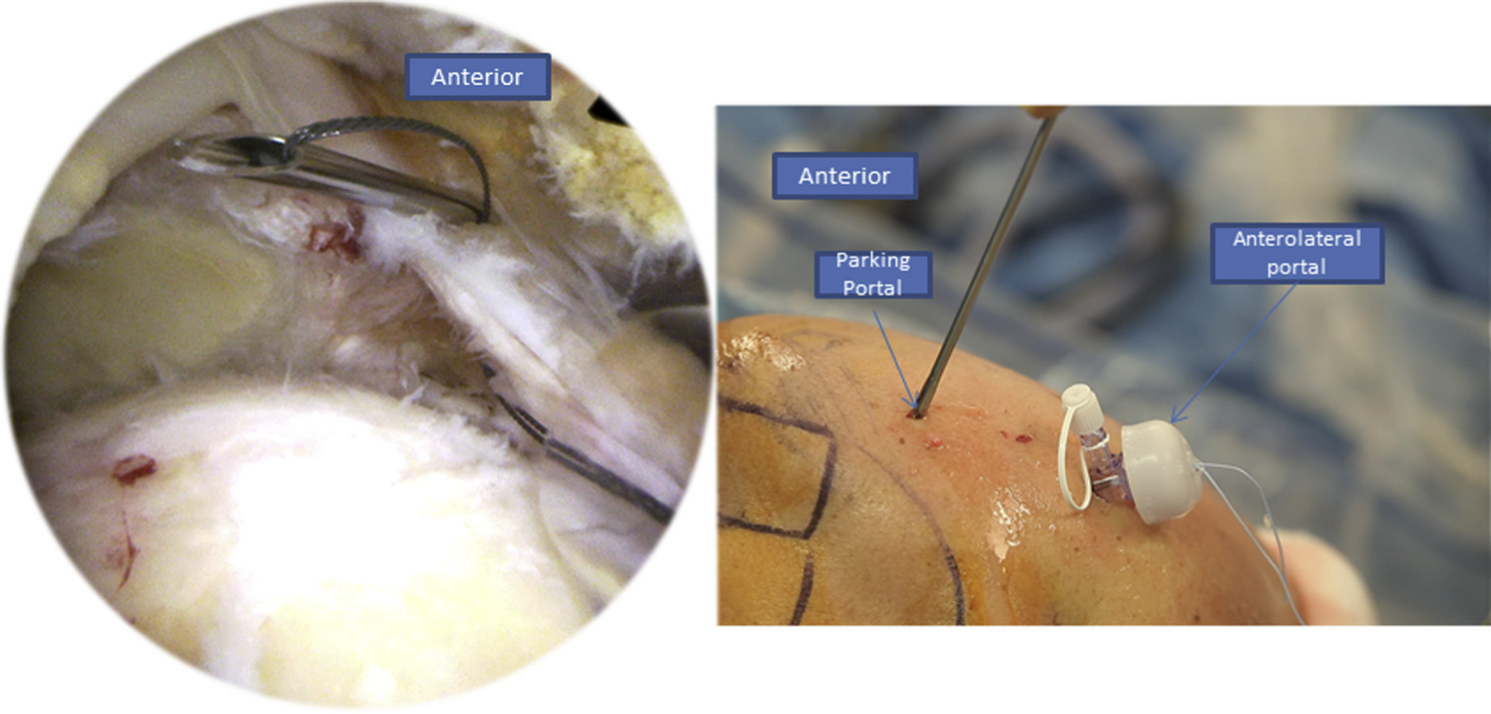
Right Side, beach chair position, posterolateral portal for the arthroscope: with grasping forceps, we retrieve the tracking wire from the parking portal.

**Fig 6:**
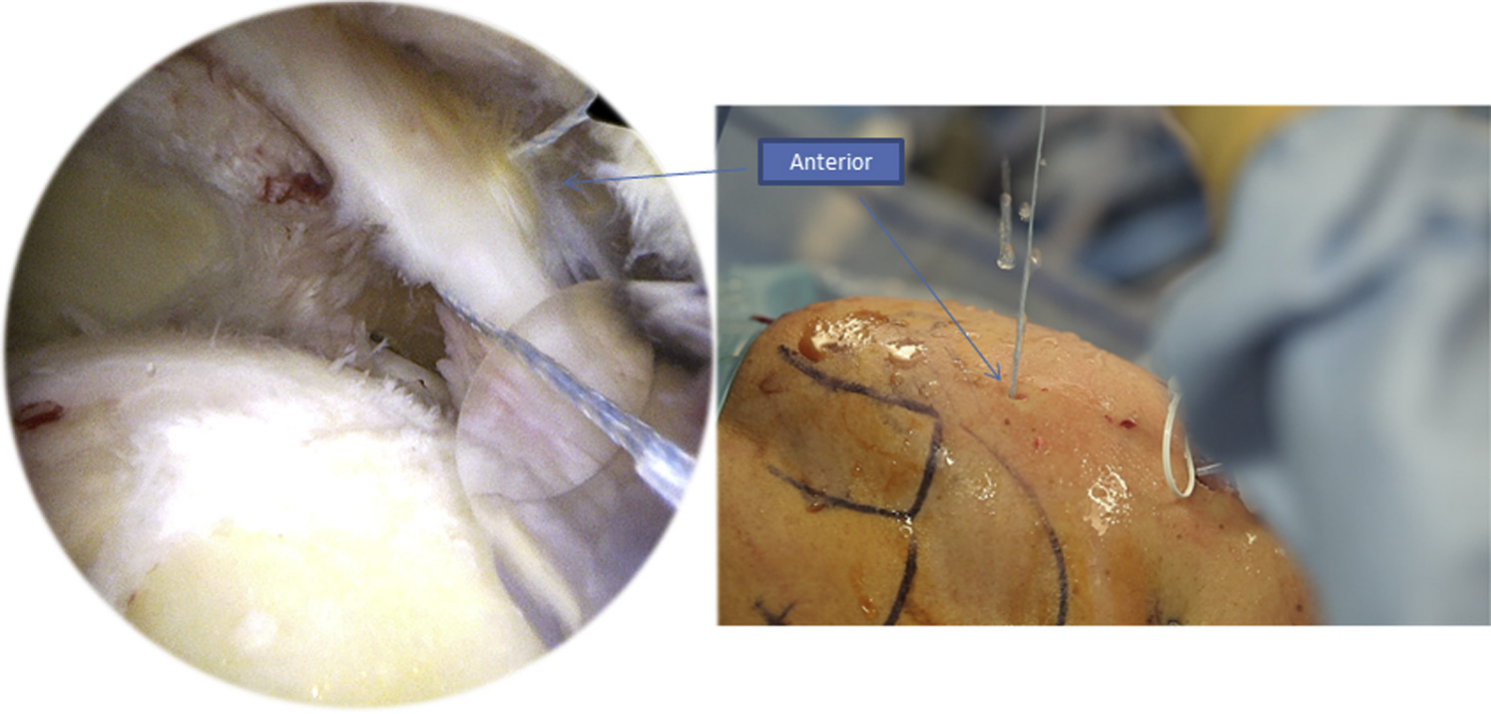
Right side, beach chair position, posterolateral portal for the arthroscope: we pull the tracking wire (Arthrex) through the parking portal to retrieve the FiberTape (Arthrex).

**Fig 7:**
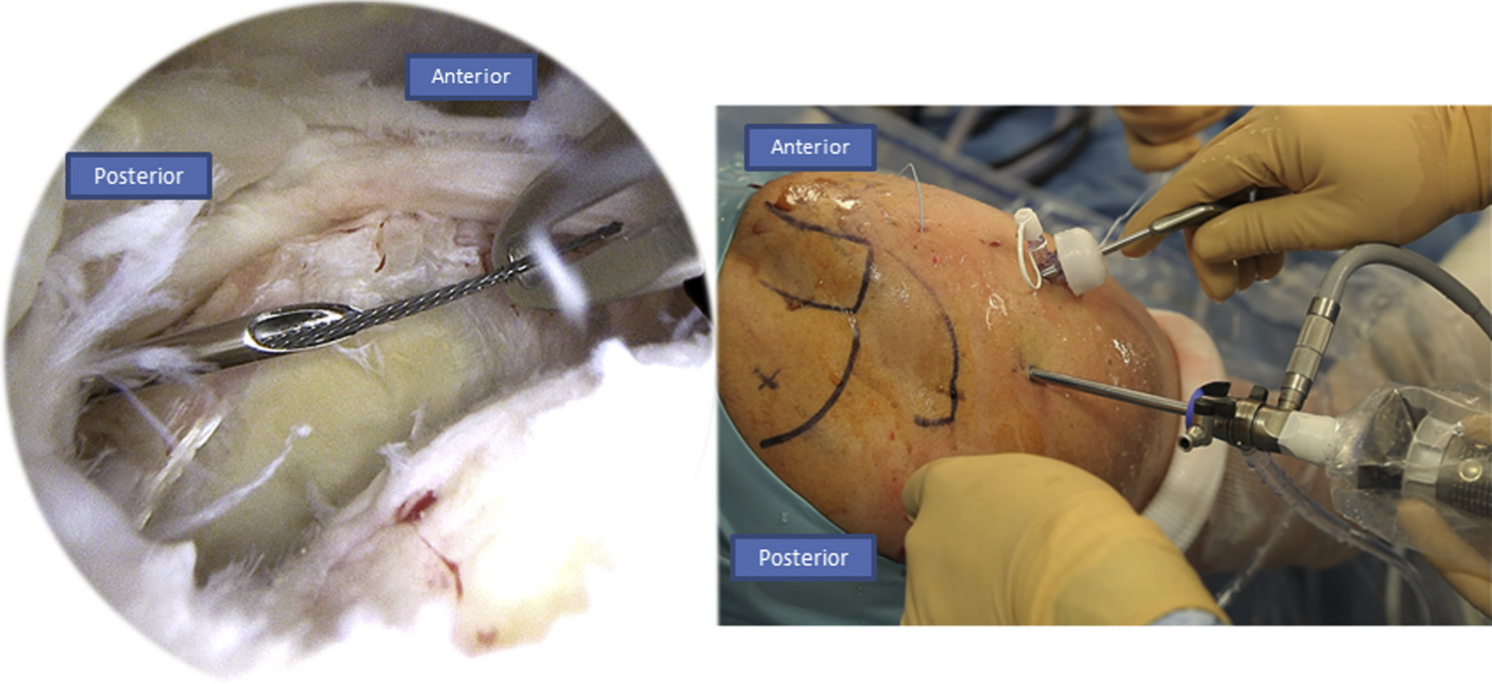
Right side, beach chair position, posterolateral portal for the arthroscope: we move to the second step of the X-suture by passing the Banana SutureLasso (Arthrex) posteriorly through the posterior portal, as you can see in the figure on the right.

We begin by introducing the Banana SutureLasso (Arthrex) anteriorly through the tear (Fig 3). We pass a tracking wire through the canula to grab the FiberTape (Arthrex) (Fig 4). Then we pass the Banana SutureLasso (Arthrex) above the tissue, and we retrieve the FiberTape (Arthrex) using the tracking wire from the deep into the superficial rotator cuff tissue and we leave it in the parking portal (Fig 5, Fig 6). We do the same posteriorly by passing the Banana SutureLasso (Arthrex) through the posterior part of the rotator cuff tear, again passing the tracking wire and retrieving the FiberTape (Arthrex) from the deep into the superficial part of the rotator cuff tear (Fig 7). The first part of the X-suture is now complete.

**Fig 8:**
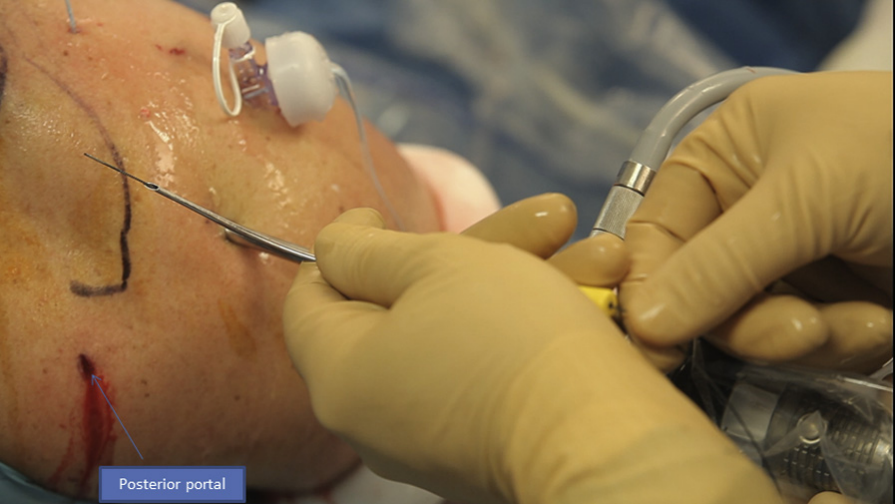
Right side, beach chair position: to begin the second part of the X-suture, we invert the loop of the tracking wire so we can pass the FiberTape (Arthrex) from the superficial into the deep part of the rotator cuff tissue.

**Fig 9:**
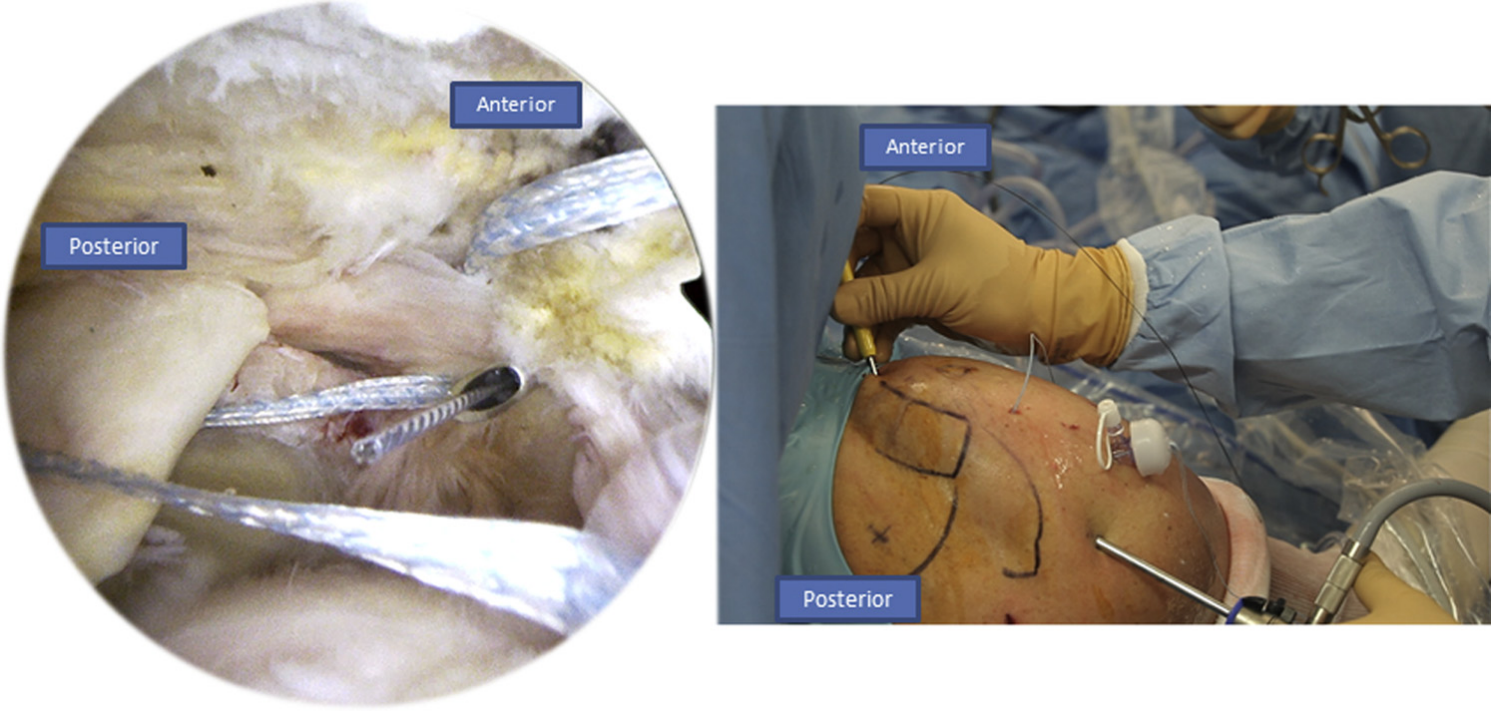
Right side, beach chair position, posterolateral portal for the arthroscope: we pass the inverted tracking wire anteriorly through the Banana SutureLasso (Arthrex).

Contrary to the first part where we retrieve the tracking wire of the Banana SutureLasso without inverting its ends, we proceed to the second part by inverting the loop of the tracking wire so we can pass from the superficial into the deep layer of the rotator cuff tissue (Fig 8). We also begin by passing through the anterior part of the tear, retrieving the FiberTape (Arthrex), and then through the posterior part while doing the same (Fig 9).

At the end we will have a complete X-suture and the FiberTape (Arthrex) is put on hold in a parking portal (Table 2).

**Table 2:** Key Steps

1. The X-suture:
• Pass the Banana SutureLasso (Arthrex) anteriorly
• Pass the tracking wire through the canula
• Pass the FiberTape (Arthrex) through the tracking wire
• Park the FiberTape (Arthrex) in the parking portal
• Do the same posteriorly
• Invert the loop of the tracking wire and pass the FiberTape (Arthrex) through the tracking wire anteriorly, then posteriorly
2. First row fixation:
• Insert a Corkscrew (Arthrex)
• Use the Banana SutureLasso (Arthrex) to pass Corkscrew (Arthrex) wires anteriorly and posteriorly
• Make half surgical knots and lock them
3. Second row fixation:
• Pass the both the FiberTape (Arthrex) and the Corkscrew (Arthrex) wires through the canula
• Place the canula on the lateral side of the humerus
• Pin down the 4 wires using a SwiveLock (Arthrex) tenodesis screw, make sure to tighten the wires to have the parachute effect before inserting the screw

### Second Part: Double-Row Fixation

**Fig 10:**
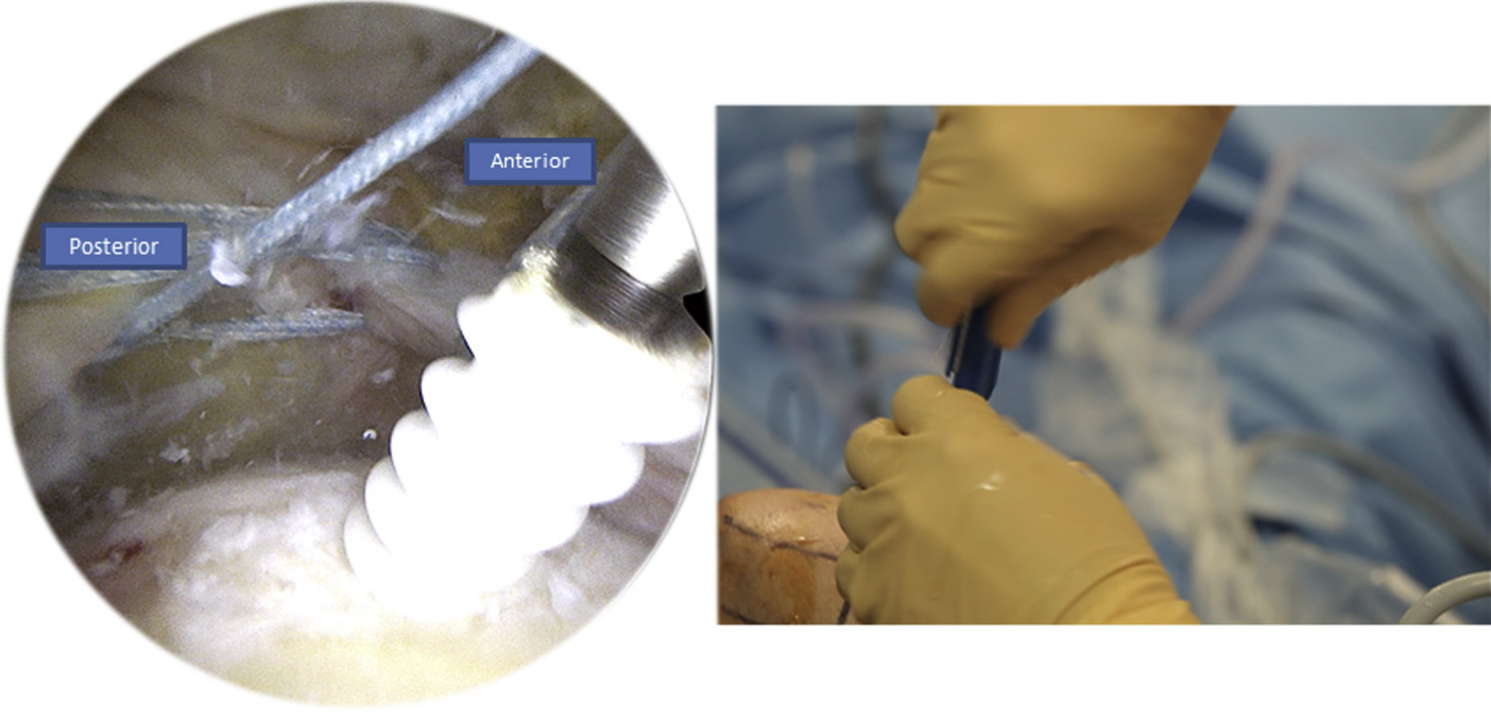
Right side, beach chair position, posterolateral portal for the arthroscope: after finishing the X-suture, we begin the double-row fixation by inserting the Corkscrew (Arthrex) through the humeral head.

**Fig 11:**
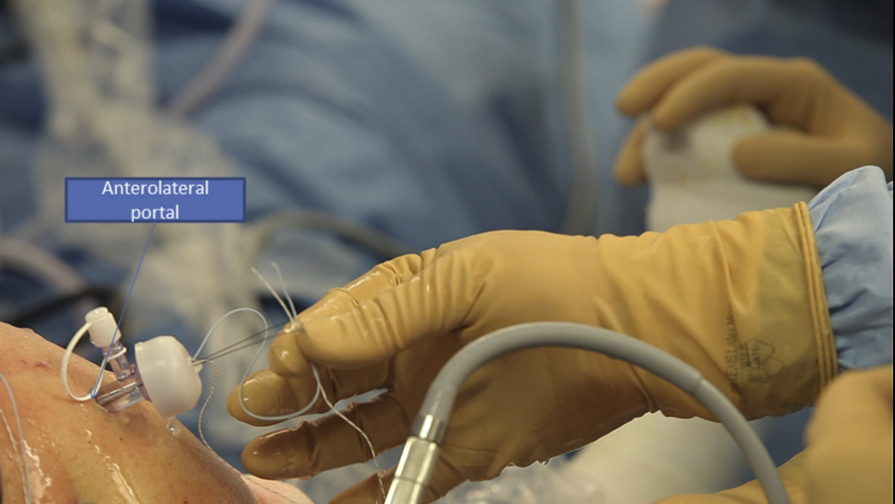
Right side, beach chair position: the Corkscrew (Arthrex) and FiberTape (Arthrex) wires are passed through the tracking wire to retrieve both of them.

After preparing the footprint using a burr or a shaver, a Corkscrew (Arthrex) is then inserted in the humeral tuberosity, close to the cartilage (Fig 10). The Corkscrew (Arthrex) and the FiberTape (Arthrex) wires are passed through the canula, and the Banana SutureLasso (Arthrex) tracking wire is passed anteriorly to retrieve both wires (Fig 11). This is what makes the originality of this technique. We do the same posteriorly by passing both wires through the posterior part of the rotator cuff tear.

**Fig 12:**
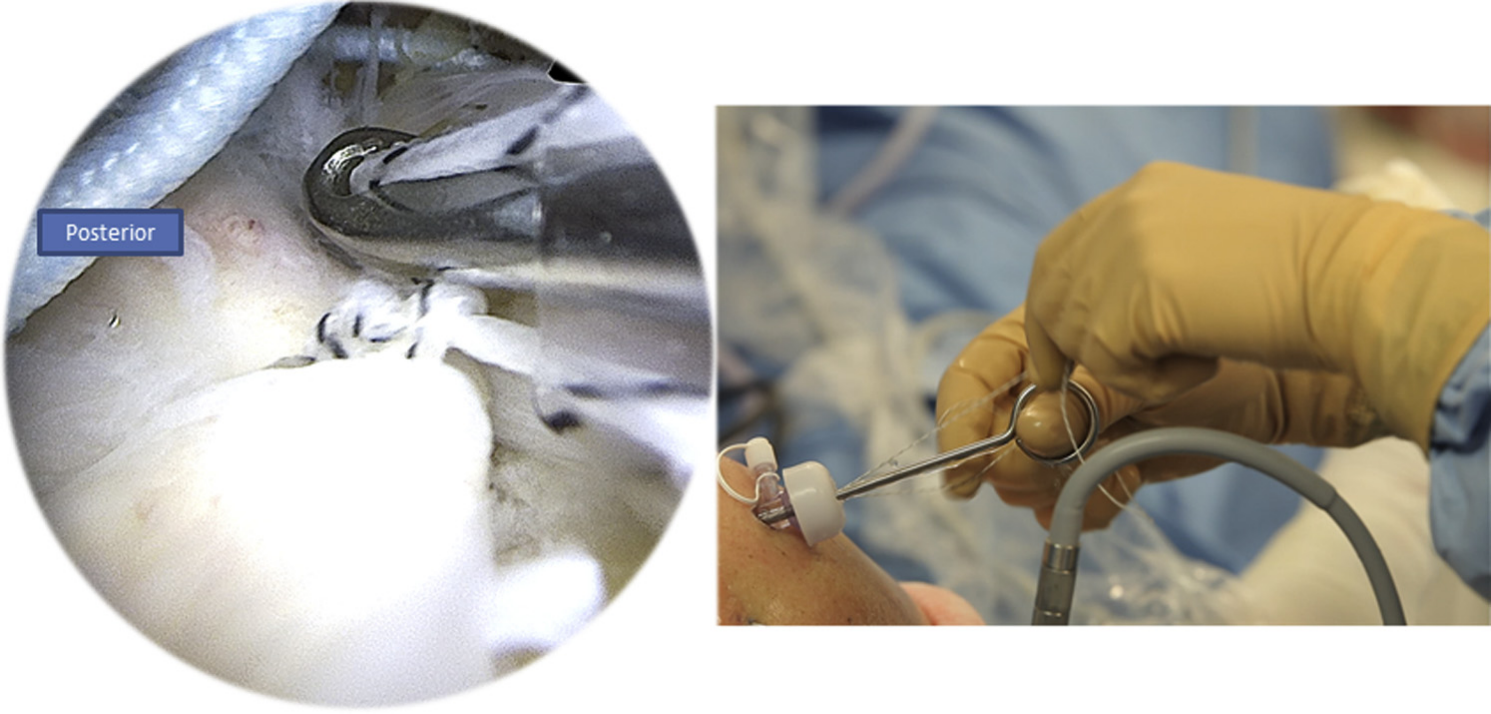
Right side, beach chair position, posterolateral portal for the arthroscope: the Corkscrew (Arthrex) wire is passed through the canula to perform half surgical hitches to repair the tear.

After passing both ends of the tear, the FiberTape (Arthrex) is put on hold in the parking portal, and the Corkscrew wire (Arthrex) is passed through the canula to perform half surgical hitches (Fig 12). Now we have a rotator cuff that is sutured and pinned down to the greater tuberosity.

**Fig 13:**
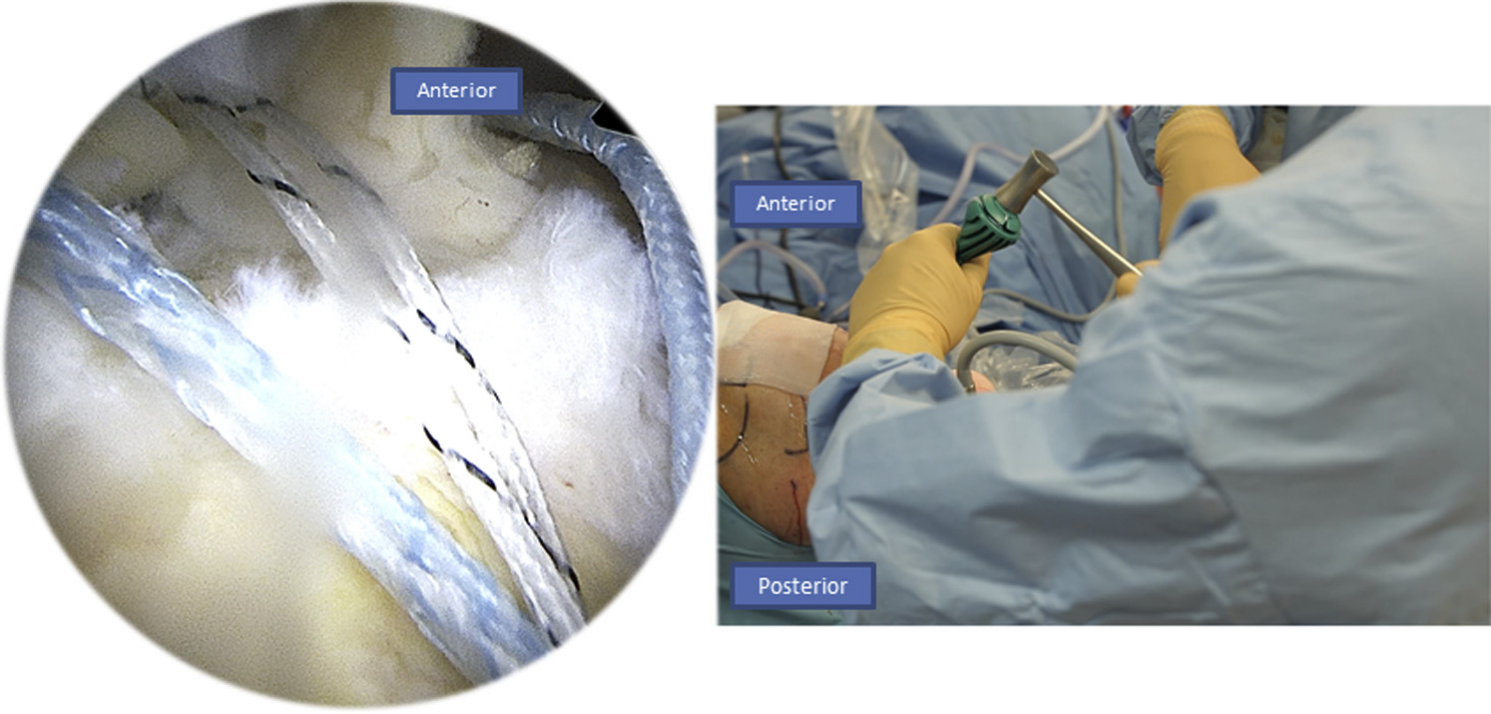
Right side, beach chair position, posterolateral portal for the arthroscope: pinning the 4 wires using a SwiveLock (Arthrex) tenodesis screw on the lateral side of the humerus.

An external rotation and abduction is performed to have a better exposure of the lateral side of the humerus. All 4 sutures are passed through the canula and through the eyelet of the SwiveLock (Arthrex) tenodesis screw (Fig 13).

**Fig 14:**
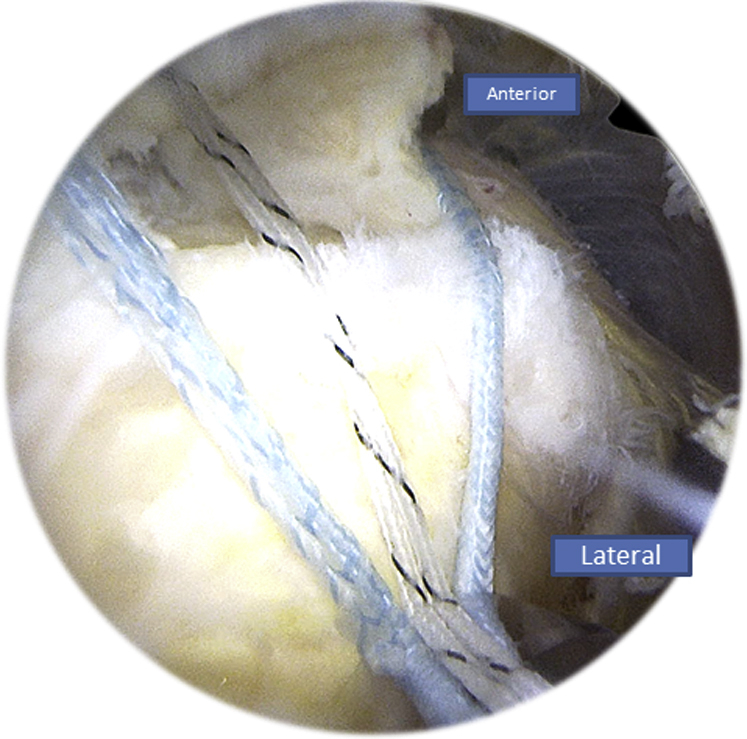
Right side, beach chair position, posterolateral portal for the arthroscope: parachute effect of the wires: the rotator cuff tissue is pinned down to the humerus using tension by the SwiveLock (Arthrex) tenodesis screw.

**Fig 15:**
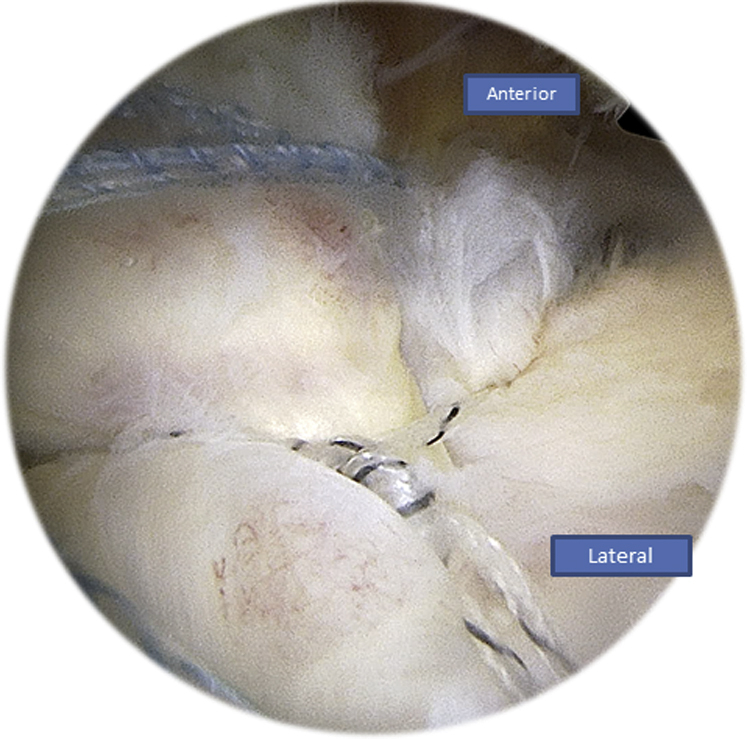
Right side, beach chair position, posterolateral portal for the arthroscope: the end result of the repair: complete coverage of the head of the humerus using an X-suture and a double-row fixation.

While tensioning the wires, we will have a parachute effect on the rotator cuff grabbing it and covering all the greater tuberosity without any tension (Fig 14, Fig 15).

### Discussion

A massive rotator cuff tear has always been a worrisome situation to every orthopaedic surgeon. Its repair demands high level of experience, agility, and arthroscopic skills. Our technique aims to simplify the complexity of the massive rotator cuff tear repair by grabbing the retracted rotator cuff tissue from its most medial part while aiming perpendicularly to it (Table 1). Another advantage includes the holding of the deep and superficial layers of the rotator cuff tissue at every suture. In that way no tension will be applied on the rotator cuff tissue while grabbing it to the greater tuberosity. Different techniques have been used including adding auto/allograft procedures. Petrie and Ismaiel^5^ found that revision rotator cuff repair with patch augmentation using human acellular dermal allograft was a safe and effective treatment method for patients with massive, retracted rotator cuff tears, but with a Banana SutureLasso (Arthrex), retracted rotator cuff tissue may be retrieved too, but further studies are required to compare the advantages of this technique over the others. Some may suggest making partial repairs, however, Heuberer et al.^6^ suggested that complete repairs were significantly superior to partial repairs and debridements, whereas debridement and partial repair did not show significant differences.

In this technique, we begin by an X-suture to join both ends of the tear so we can have a single mattress to repair and to have a better hold on the retracted tissue. To do so, we have to find the right entry point of the Banana SutureLasso (Arthrex); this requires a learning curve like in every technique. Another limitation includes the adjustment of the wires at every single step (Table 1).

After that, we use a double-row technique to pin down the tissue to the greater tuberosity and adding a parachute effect on the lateral side of the humerus to cover all of the head of the humerus. Although there are no studies confirming the superior advantage in clinical outcome scores in all types of repairs, controversy still exists concerning the indication of the double- versus single-row techniques in massive rotator cuff repairs. Carbonel et al.^7^ reported that a significant improvement with the double-row technique was shown in clinical evaluation, and that the improvements were more significant in tears greater than 30 mm than in 10- to 30-mm tears.

## Conclusions

Using this technique has simplified massive rotator cuff tear repairs and offered an easier approach, and maybe more indications for the years to come. It is simple and reproducible, does not cost more, and offers a better chance of repair compared with the others. Further studies need to be done to ensure the safety and importance of this technique.

## Supplementary Data

Video 1 The patient is at a beach chair position. The scope and the shaver are introduced laterally just over the tear. The first step is to have an interrupted X-suture. This why we introduce the Banana SutureLasso anteriorly through the tear. We use the tracking wire in order to pass the fibertape from the deep into the superficial layer of the rotator cuff tissue. Then we leave the fibertape in the parking portal just over the tear. We do the same posteriorly; you can see how the Banana Lasso passes through both the superficial and the deep layers of the rotator cuff tear, something you can't do while using the scorpion forceps. Note how easily the fibertape is passed while using the tracking wire. The first part of the X-suture is now complete. Contrary to it, we proceed to the second part by inverting the loop of the tracking in order to pass the fibertape from the superficial into the deep layer of the rotator cuff tissue. In that way enough tissue is taken and no tension will be applied while tying the knots. We do the same on the posterior part, also by inverting the loop of the tracking wire so we can have a complete X-suture. The tracking wire is then passed through the canula and the fibertape is inserted through the tear and again put on hold in the parking portal. With this X-suture technique, using the Banana Lasso, we have enough tissue taken medially to ensure a better fixation with no tissue laceration. After preparing the footprint, a corkscrew is then inserted in the humeral tuberosity, to begin the double row fixation. The corkscrew and the fibertape wires are taken together with a grasping forceps through the canula and this what makes the originality of this technique. Then again, the Banana Lasso is inserted anteriorly, this time the tracking wire holding both the fibertape and the corkscrew wires are again passed anteriorly from the deep into the superficial layer of the anterior of the rotator cuff tear. We do the same posteriorly by passing the 2 wires from the deep into the superficial layer of the posterior part of the rotator cuff tear. After passing both ends of the tear, the fibertape wire is put on hold in the parking portal while the corkscrew wire is passed through the canula and is tightened with half surgical hitches so we can slide them and then lock them with an inverted half hitch. Now we have a rotator cuff that is sutured and pinned down to the greater tuberosity. An external rotation and abduction is performed in order to have a better exposure of the lateral side of the humerus. All 4 sutures are passed through the canula and through the eyelet of the swivelock screw, then pinned down to the lateral side of the humerus. You can see the parachute effect of the wires on the rotator cuff, grabbing it and covering all the head of the humerus without any tension.

ICMJE author disclosure forms
